# Preconception origins of perinatal maternal mental health

**DOI:** 10.1007/s00737-020-01096-y

**Published:** 2021-01-23

**Authors:** Michelle Z. L. Kee, Santhi Ponmudi, Desiree Y. Phua, Anne Rifkin-Graboi, Yap Seng Chong, Kok Hian Tan, Jerry Kok Yen Chan, Birit F.P. Broekman, Helen Chen, Michael J. Meaney

**Affiliations:** 1 Translational Neuroscience Program, Singapore Institute for Clinical Sciences, A*STAR, Singapore, Singapore; 2 Centre for Research in Child Development, Office of Educational Research, National Institute of Education, Nanyang Technological University, Singapore, Singapore; 3 Yong Loo Lin School of Medicine, National University of Singapore, Singapore, Singapore; 4 Department of Reproductive Medicine, KK Women’s and Children’s Hospital, Singapore, Singapore; 5 Department of Maternal Fetal Medicine, KK Women’s and Children’s Hospital, Singapore, Singapore; 6 Department of Psychiatry, OLVG and Amsterdam UMC (location VUmc), VU University, Amsterdam, The Netherlands; 7 Department of Psychological Medicine, KK Women’s and Children’s Hospital, Singapore, Singapore; 8 Sackler Program for Epigenetics & Psychobiology at McGill University, Montreal, Canada; 9 Department of Psychiatry, Douglas Mental Health University Institute, McGill University, Montreal, Canada

**Keywords:** Preconception, Pregnancy, Maternal mental health, Depressive symptoms, Anxiety symptoms

## Abstract

Perinatal maternal symptoms of depression and anxiety compromise psychosocial function and influence developmental outcomes in the offspring. The onset of symptoms remains unclear with findings that suggest a preconceptual origin. We addressed this issue with a prospective analysis of anxiety and depressive symptom profiles from preconception through to parturition. Women were recruited into a preconception study to assess (a) variation in symptom levels of depression and anxiety from pre- to post-conception and (b) if the symptom network profiles of depression and anxiety change from pre-conception to post-conception. A within-subject intraclass correlation analyses revealed that symptoms of depression or anxiety in the preconception phase strongly predicted those across pregnancy and into the early postnatal period. The symptom network analysis revealed that the symptom profiles remained largely unchanged from preconception into the second trimester. Our findings suggest that for a significant portion of women, maternal mental health remains stable from preconception into pregnancy. This finding highlights the need for early intervention studies on women’s mental health to be targeted during the preconception period and to be extended across the population.

## Introduction

The incidence of peripartum depression in economically developed countries is generally between 10 and 12%, with markedly higher rates in low- and middle-income countries (Gavin et al. 2005; Wachs et al. 2009; Sawyer et al. 2010). Despite the common use of the term “post-partum depression,” it is well established that the onset of clinical levels of depressive symptoms can occur during both the antenatal and postnatal periods (Gavin et al. 2005; Milgrom et al. 2008; Wilcox et al. 2020). Accordingly, the most recent edition of the Diagnostic and Statistical Manual of Mental Disorders (DSM-V) expanded the definition of peripartum depression to include onset of symptoms during pregnancy and for up to 4 weeks postpartum (American Psychiatric Association 2013), although experts are divided in the consensus on the timing of onset (Postpartum Depression: Action Towards Causes and Treatment (PACT) Consortium 2015).

The intense public health focus on maternal mental health is justified in terms of the well-being of the mother as well as the impact on the offspring. An extensive economic analysis (Bauer et al. 2014) reveals that 72% of the health care costs associated with poor maternal mental health were linked to the offspring. Longitudinal analyses reveal that the quality of parent–child interactions over the postnatal period mediates associations between maternal mental health and child outcomes (Pawlby et al. 2011; Kok et al. 2013; Plant et al. 2015; Edwards and Hans 2016; Bouvette-Turcot et al. 2019). Moreover, the effective postnatal treatment of maternal depression improves outcomes for the offspring (Weissman et al. 2016; Stein et al. 2018). There is also compelling evidence for antenatal maternal depression influencing child health and development. Neonatal neuroimaging studies confirm the direct association between the quality of antenatal maternal mental health and brain structure and connectivity of the offspring at birth (Buss et al. 2012; Sandman et al. 2015; Rifkin-Graboi et al. 2013; Qiu et al. 2015, 2017; Dean et al. 2018). Epidemiological analyses likewise document associations between antenatal maternal mental health and socio-emotional function, academic achievement, and the later risk for psychopathology in the offspring independent of postnatal maternal mental health (e.g., Evans et al. 2011; Cents et al. 2013; Stein et al. 2014; Pearson et al. 2016). In sum, studies of child development suggest that maternal mental health operates over the perinatal period to influence child outcomes.

More recent studies also reveal the impact of high, sub-clinical symptom levels of depression (Meaney 2018 for a review). Importantly, women with high, sub-clinical levels are as affected in terms of psychosocial function and parenting as are those with clinical levels of depressive symptoms (Judd et al. 1996; Weinberg et al. 2001). Estimates of the impact of maternal mental health problems based on clinical cut-offs or diagnosis thus underestimate the extent of the problem across the population. Collectively, mothers with clinical and high-subclinical levels of depressive symptoms (i.e., EPDS scores of 9 or 10 and above) include as much as 40% of the population in economically developed countries, thus representing a public health issue of remarkable importance (Meaney 2018). Moreover, while less well studied, the prevalence rates of perinatal maternal anxiety appear even higher than those for depression, and consequences for the development of the offspring are at least as impactful as are those of depressive symptoms (Van Batenburg-Eddes et al. 2013; Glover 2014; O’Donnell and Meaney 2017; Dean et al. 2018; Madigan et al. 2018). Antenatal maternal anxiety predicts variations in neural structures related with cognitive-emotional response to stress, sensory processing, and socio-emotional function in neonates at 5–17 days postnatal (Rifkin-Graboi et al. 2015; Qiu et al. 2015).

The literature on the incidence of depression over the perinatal period is mixed with reports from one systematic review revealing somewhat higher levels during the antenatal period (e.g., Gavin et al. 2005) and an another reporting the opposite trend (Bennett et al. 2004). However, longitudinal trajectory analyses reveal that depressive symptom levels are largely stable over the ante- and post-natal periods (Santos et al. 2017; Lim et al. 2019) with similar profiles for perceivied stress (Lim et al. 2020). Analysis of the large Avon Longitudinal Study of Parents and Children (ALSPAC) cohort reveals modestly higher symptom levels in early pregnancy (Evans et al. 2001; Heron et al. 2004) and a comparable trend for symptoms of anxiety (Heron et al. 2004). Latent class analyses and other forms of modeling using longitudinal cohort data consistently show that most women exhibit stable low, moderate, and high symptoms levels of depression (Cents et al. 2013; Giallo et al. 2014; Van Der Waerden et al. 2015; Park et al. 2018; see Santos et al. (2017) for an extensive review), with a smaller percentage of women an increase in symptoms that reached clinical levels post-partum and showing a post-partum onset of clinical levels of symptoms (but also see Postpartum Depression: Action Towards Causes and Treatment (PACT) Consortium 2015). However, there are mixed reports on the stability of anxiety levels during pregnancy and postpartum. Whilst most studies observe lower levels of anxiety after delivery than during pregnancy (Heron et al. 2004; Figueiredo and Conde 2011), others reveal no difference in anxiety levels (Grant et al. 2008). Thus, while some women experience a post-partum onset of depression, the evidence suggests that most women generally report stable levels of depressive and anxiety symptoms over the peripartum period.

The relative stability of maternal symptoms of depression and anxiety is not surprising since the strongest predictor of postnatal depression is depression in pregnancy (O’Hara and Swain 1996; Llewellyn et al. 1997; Leigh and Milgrom 2008). The origins of clinical or high, sub-clinical maternal symptoms of depression and anxiety may extend even further into the preconception period. Marcus et al. (2003) found that almost half of the women depressed during pregnancy had a history of major depressive disorder. The most important risk factor for perinatal depression is that of a prior history of depression (Leigh and Milgrom 2008; Giallo et al. 2014; Patton et al. 2015). Perinatal mental health problems are commonly preceded by problems prior to conception often in adolescence (Patton et al. 2015). Reviews of psychosocial risk factors for perinatal depression reveal strong evidence for social support, domestic violence, stress, and low socio-economic status (Lancaster et al. 2010; Yim et al. 2015; Biaggi et al. 2016; Santos et al. 2017). These same factors also predict depressive and anxiety disorders in the general population (Clark et al. 1994; Rijsdijk et al. 2001; Stein et al. 2014; Wang et al. 2018). Stable personality traits such as neuroticism that predict anxiety and depression in the general population also predict the risk for poor maternal mental health (Leigh and Milgrom 2008; Martini et al. 2015; Denis and Luminet 2018). Measures of social support and self-esteem that diminish the risk for depression and anxiety in the general population have a comparable influence on the risk for peripartum depression and anxiety (Elsenbruch et al. 2007; Leigh and Milgrom 2008; Martini et al. 2015). Finally, though we lack a comprehensive analysis of genetic variations and maternal mental health, the existing studies, while largely limited to studies of candidate genes, reveal that polymorphisms previously associated with major depressive disorder are implicated in perinatal depression (Doornbos et al. 2009; Binder et al. 2010; Comasco et al. 2011; Mehta et al. 2012; Alvim-Soares et al. 2013).

Taken together, these findings suggest that maternal symptoms of depression may predate conception. Spry et al. (2019) provided striking evidence for this idea in a prospective study revealing that preconception maternal depression predicted emotional reactivity of the offspring independent of depression in either the antenatal or postnatal period. The issue is of considerable importance for public health. The relevant timing for effective screening and intervention studies would be greatly affected if indeed high symptom levels of depression or anxiety are apparent prior to conception.

In the following study, we used a preconception cohort study in which women were recruited prior to conception to examine symptoms of both depression and anxiety from the preconception period into the antenatal and early postnatal period. We hypothesized that (a) symptoms of depression or anxiety are highly stable across preconception, pregnancy and into the post-partum period, and (b) the symptom profiles using symptom network analyses would remain largely unchanged from preconception to mid-gestation.

## Materials and methods

### Cohort description

This study is based on data from the Singapore PREconception Study of long-Term maternal and child Outcomes (S-PRESTO). Women who were planning a pregnancy were recruited from a general population between February 2015 and October 2017 through notices describing the S-PRESTO project that were posted in relevant clinical settings and on social media. Women aged 18 to 45 years and of either Chinese, Malay, Indian, or any combination of these three ethnicities were included in the cohort. Exclusion criteria preclude recruitment of women who (1) were diagnosed with Type 1 or Type 2 diabetes mellitus, (2) had received assisted fertility treatment or taken contraceptives in the past month, or (3) had received systemic steroids, anticonvulsants, HIV or hepatitis B or C medications in the past month. Women (*N* = 1709) expressing an interest in the S-PRESTO project were approached and 1032 (60.4%) were consented and recruited into the study. Written informed consent was obtained from all participants. Ethical approval was obtained from the SingHealth Centralized Institutional Review Board (reference 2019/2143) and the tenets of the Declaration of Helsinki were observed.

### Measures of mental well-being

Anxiety and depressive symptoms were assessed by questionnaires administered once every 3 months preconception (PC1, PC2 and PC3) and over pregnancy. The first trimester of pregnancy (PG1) was defined as 3–12 weeks of gestation, the second trimester (PG2) as 13–28 weeks of gestation, and the third trimester (PG3) as 28–40+ weeks of gestation. Questionnaires were also administered 3 months post-delivery. Anxiety symptoms were assessed using the Spielberger State-Trait Anxiety Inventory (STAI; Spielberger 1983). The STAI consists of 40 items with a 4-point Likert scale assessing both transient (“state” measure) and stable (“trait” measure) characteristics of anxiety. By calculating for Cronbach’s alpha, high internal consistency was observed at each time point for STAI-state (0.93–0.95) and STAI-trait (0.92–0.95). The scores for STAI were reversed scored and summed. All STAI scores were analyzed in a continuous manner. Depressive symptoms were assessed using the Edinburgh Postnatal Depressive Scale (EPDS; Cox et al. 1987). The EPDS is a validated self-report instrument that contains 10 items of common depressive symptoms over the past week. The EPDS is sensitive and reliable in detecting both antenatal depression in women (Matthey et al. 2006) as well as postnatal depression. We used the EPDS to categorize levels of depression during preconception, antenatally and postnatally according to the following cut-offs: 0–4 (low), 5–8 (mild), 9–13 (high, subclinical), and ≥ 14 (clinical). We decided to use these same cut-offs to explore the stability of EPDS across preconception, antenatal and post-partum, although Matthey et al. (2006) suggested different cut-offs during pregnancy (≥ 15) and post-partum (≥ 13). Cronbach’s alpha for the EPDS at each given time was calculated and ranged from 0.83 to 0.88, showing high internal consistency of the EPDS.

### Socio-demographic measures

Information on age, highest education level attained, and marital and employment status were obtained during the first clinic visit.

### Statistical analyses

**Fig. 1:**
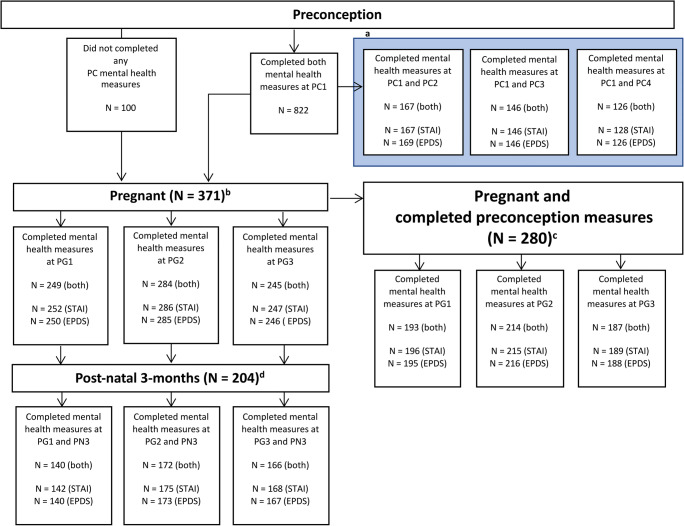
Flowchart describing participation of women in the preconception and pregnancy period. Mental health measures here refers to both the STAI and EPDS measures. Data were used to calculate intraclass correlations during ^a^ preconception (PC), ^b^ pregnancy (PG), and ^d^ between pregnancy and 3 months post-delivery (PN3) (Fig. 2). ^c^ Data was used to calculate intraclass correlations between PC and PG timepoints in Fig. 2, Fig. 3, and Table 2

Descriptive statistics were generated for the whole SPRESTO cohort and for participants who completed the instruments related to mental health at first preconception visit. Eight hundred twenty-two out of 1032 participants recruited for the SPRESTO cohort completed questionnaires on their mental well-being (the STAI and EPDS) at the first preconception visit (PC1). A subset of these women was asked to complete both mental health measures every 3 months during the preconception period until they were pregnant, at which point they completed questionnaires at every trimester (Fig. 1). All 1032 subjects recruited were also asked to complete the mental health measures during each pregnancy trimester. Three hundred seventy-one pregnant subjects completed at least one of the mental health measures, while 26 pregnant subjects did not complete any of the mental health measures. Pearson’s chi-square tests, two-sided Fisher’s exact test, and independent *t* test were used to compare differences between these two groups of subjects. Internal consistency for each questionnaire related to mental health was measured by Cronbach’s alpha. Test-retest reliability of the two tests was assessed by intra-class correlation coefficient (ICC) using single-measurement, absolute-agreement, two-way mixed-effects model with 95% confidence interval. Based on Koo and Li (Koo and Li 2016), ICC estimates below 0.50 were considered to have poor test-retest reliability; 0.50–0.75 as moderate; 0.75–0.90 as good; and above 0.90 as excellent. Data are medians unless otherwise stated. These statistical analyses were conducted in SPSS (Version 24.0, IBM Corporation, Armonk, NY).

Pairwise deletion of subjects was performed for each of the subsequent analyses. Wilcoxon signed-rank was used to compare the differences between EPDS scores at two time-points respectively. A paired McNemar-Bowker chi-square test of symmetry was performed to compare the EPDS group differences between preconception and the trimesters of pregnancy in R 3.6.0 (R Core Team 2019). Post hoc analyses were performed with false discovery rate (FDR) correction.

### Network analyses

Depressive and anxiety symptoms’ networks were estimated separately on subjects who conceived within a year, using the Fused Graphical Lasso (FGL) technique via the EstimateGroupNetwork R package (Danaher et al. 2014; Epskamp et al. 2018). The preconception and prenatal networks were estimated jointly in FGL and the joint estimation improves network estimates by considering similarities between the two networks. The polychoric correlation matrices of the preconception and prenatal depressive (and anxiety) networks were computed separately. These matrices were then used to estimate the preconception and prenatal networks simultaneously (Constantini et al. 2020). The networks were visualized using the qgraph R package (Epskamp et al. 2012). The thickness of the edges illustrates the strength of the partial correlation between two symptoms.

We examined the change in network structure, global connectivity, and influential symptoms before and during pregnancy. Influential symptoms were measured using the expected influence (EI) index. EI indices indicate the strength of connections each symptom has to other symptoms, taking the directionality of the connections into account. Changes in symptoms with higher EI are more likely to activate (or deactivate) other symptoms within the network as they are stronger and more positively connected to other symptoms (Robinaugh et al. 2016). EI was estimated via the qgraph package (Epskamp et al. 2012) and the temporal stability of the EI indices was measured by Spearman correlation test. The reliability of the EI indices and network parameters were assessed via the bootnet package using 10,000 bootnet samples (Epskamp et al. 2018). Changes in network structure, global connectivity, and symptom EI were assessed using 10,000 permuted networks via the NetworkComparisonTest R package (van Borkulo 2018).

## Results

### Study characteristics

Demographic characteristics between pregnant women who completed and did not complete the mental health measures are summarized in Table 1. Pregnant women who completed the mental health measures during pregnancy and those who did not complete the measures during pregnancy were of similar age (*t* (337) = −0.30, *p* = .82). The groups of participants also did not differ in terms of highest education levels attained (*χ*^2^_(2)_, = 0.68, *p* = .71), ethnicity (*χ*^2^_(2)_, = 0.45, *p* = .80), marital status (*χ*^2^_(3)_, = 1.15, *p* = .76), or employment status (*p* = .36; two-tailed Fisher’s exact test).

**Table 1:** Study characteristics

Variables	With mental health data (*N* = 371)	Without mental health data (*N* = 26)	*P* value
	Means ± SDs/N (%)*	Means ± SDs/ N (%)*	
Age at enrolment (years)	30.7 ± 3.2	31.0 ± 4.3	.82
Ethnicity			.80 ^a^
Chinese	295 (79.5)	22 (84.6)	
Indian	24 (6.5)	1 (3.8)	
Malay	52 (14.0)	3 (11.5)	
Marital status			.76 ^a^
Never married	5 (1.3)	1 (3.8)	
Married	364 (98.1)	25 (96.2)	
Separated	1 (0.3)	–	
Divorced	1 (0.3)	–	
Highest education level			.71 ^a^
Primary/secondary	10 (2.7)	1 (3.8)	
Post-secondary	91 (24.5)	8 (30.8)	
Tertiary and above	270 (72.8)	17 (65.4)	
Employment status			.36 ^b^
Unemployed	46 (12.4)	5 (19.2)	
Employed	324 (87.3)	21 (80.8)	
Missing	1 (0.3)	–	

*P* values are based on ^a^Pearson’s chi-square tests or ^b^two-sided Fisher’s exact test for categorical variables, and independent *t* tests for continuous variables. *Percentages are rounded off to nearest 0.1%

### Symptoms of depression and anxiety

#### Preconception period

**Fig. 2:**
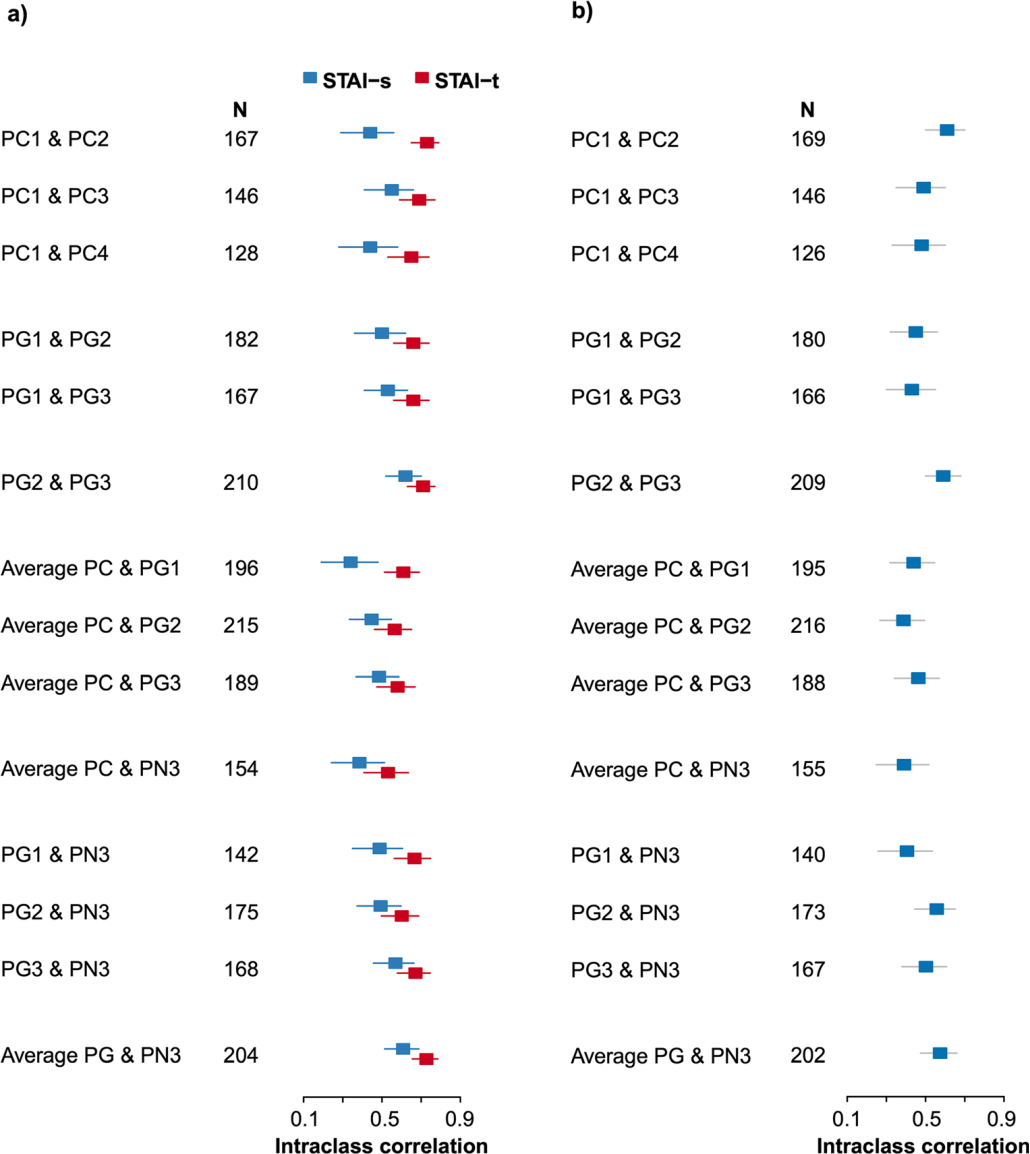
Intraclass correlations of (a) STAI-state (STAI-s), STAI-trait (STAI-t), and (b) EPDS, across two or three timepoints during preconception (PC), pregnancy (PG), and 3 months post-delivery (PN3). Each PC and PG timepoints were separated by 3-months interval. Forest plot with 95% confidence intervals for the estimates of the intraclass correlations

We examined the corresponding of levels of symptoms of depression and anxiety over the periconceptual period comparing preconception and pregnancy scores. Our objective was to be able to detect a meaningful change in mental health from pre- to post-conception against the background of normally occurring variation in the self-reports of symptoms over time within the preconception period (i.e., test/re-test variation). Intra-class correlations (ICC) were calculated for the EPDS, STAI-state, and STAI-trait using measures obtained across the preconception period and separated by 3-month intervals. STAI-state scores between any two time points during preconception had poor to moderate concordance (ICC = 0.44–0.55; 95% CI = 0.28–0.66) (Fig. 2a). This was expected as STAI-state examines current symptoms that can vary dynamically with time. Conversely under the same conditions, STAI-trait had higher concordance at a moderate level (ICC = 0.53–0.73), with a smaller 95% CI range of 0.53–0.79. Similar to STAI-trait, EPDS scores were also moderately stable when taken between PC1 and PC2 (ICC = 0.61, 95% CI = 0.5–0.7) (Fig. 2b). However, EPDS scores showed poorer concordance when taken more than 3 months apart during preconception (ICC = 0.48–0.49; 95% CI = 0.33–0.6) (Fig. 2b).

#### Preconception to pregnancy

The primary focus of our analyses was to compare symptom levels from the pre-conception to post-conception period. For this comparison, we calculated an “average preconception symptoms score” using each of the available preconception time points before performing ICC. Despite having poor concordance in STAI-state scores between preconception and multiple periods across pregnancy (ICC = 0.34–0.49), the ICC remained within the 95% CI range of ICC during preconception period (Fig. 2a). Visual analysis of the ICC’s calculated across the multiple periconceptual periods for STAI-trait and EPDS also revealed that the ICC’s for the average preconception score and each of the 3 trimesters of pregnancy (PG1, PG2 and PG3) fell well within the range of those during preconception (Fig. 2). Hence, the variation across the critical pre-conception to post-conception periods did not exceed the statistical noise associated with test-re-test reliability.

We then compared symptom scores across pregnancy. There was no significant variation in STAI-state scores across the three trimester time points (χ^2^_(2)_ = 3.71, *p* = .16). There were very modest but statistically reliable changes in symptom scores when comparing between individual trimesters. STAI-state score was significantly higher in the first (37.9 ± 10.0) compared to the second trimester (34 ± 10.2; *z* = − 4.61, *p* < .001; *N* = 182). For the 167 participants who completed STAI in both PG1 and PG3, the STAI-state score was also significantly higher in the first (37.4 ± 10.0) compared to the third trimester (35.5 ± 10.6; *z* = − 2.23, *p* = .03). However, when comparing within participants who completed STAI at both PG2 and PG3, STAI-state scores significantly increased from second (32.7 ± 9.7) to third trimester (34.5 ± 10.3; *z* = − 3.31, *p* = .001, *N* = 210). Similarly, there was no significant change in STAI-trait scores observed across the three trimesters as well (*χ*^2^_(2)_ = 3.00, *p* = .22). When comparing within participants who completed EPDS in both PG1 and PG2, we found a small but reliable decrease in STAI-trait scores from the first (38.5 ± 9.4) to second trimester (36.4 ± 9.2; *z* = −4.10, *p* < .001, *N* = 182). STAI-trait scores also decreased slightly but significantly from the first (38.0 ± 9.6) to third trimester (36.4 ± 9.5; *z* = − 2.74, *p* = .01, *N* = 167). EPDS scores showed the same profile with no significant change across trimester time points (*χ*^2^_(2)_ = 3.00, *p* = .22), but with higher scores in the first (7.1 ± 4.6) compared to the second trimester (6.4 ± 4.7; *z* = 2.55, *p* = .01; *N* = 180).

### Depressive symptoms from preconception to pregnancy

The EPDS is a screening measure with clinically-validated cut-offs allowing for assessment of “probable” clinical categorical status over time (Matthey et al. 2006). Since women with high, sub-clinical levels of depressive symptoms do not differ from those with clinical levels in measures of psychosocial impairments (Judd et al. 1996; Weinberg et al. 2001), we considered these women to be within a single category. We then discerned the percentage of women in the clinical/high-subclinical category in each trimester, who were similarly categorized in the preconception period (using the average preconception EPDS score).

**Fig. 3:**
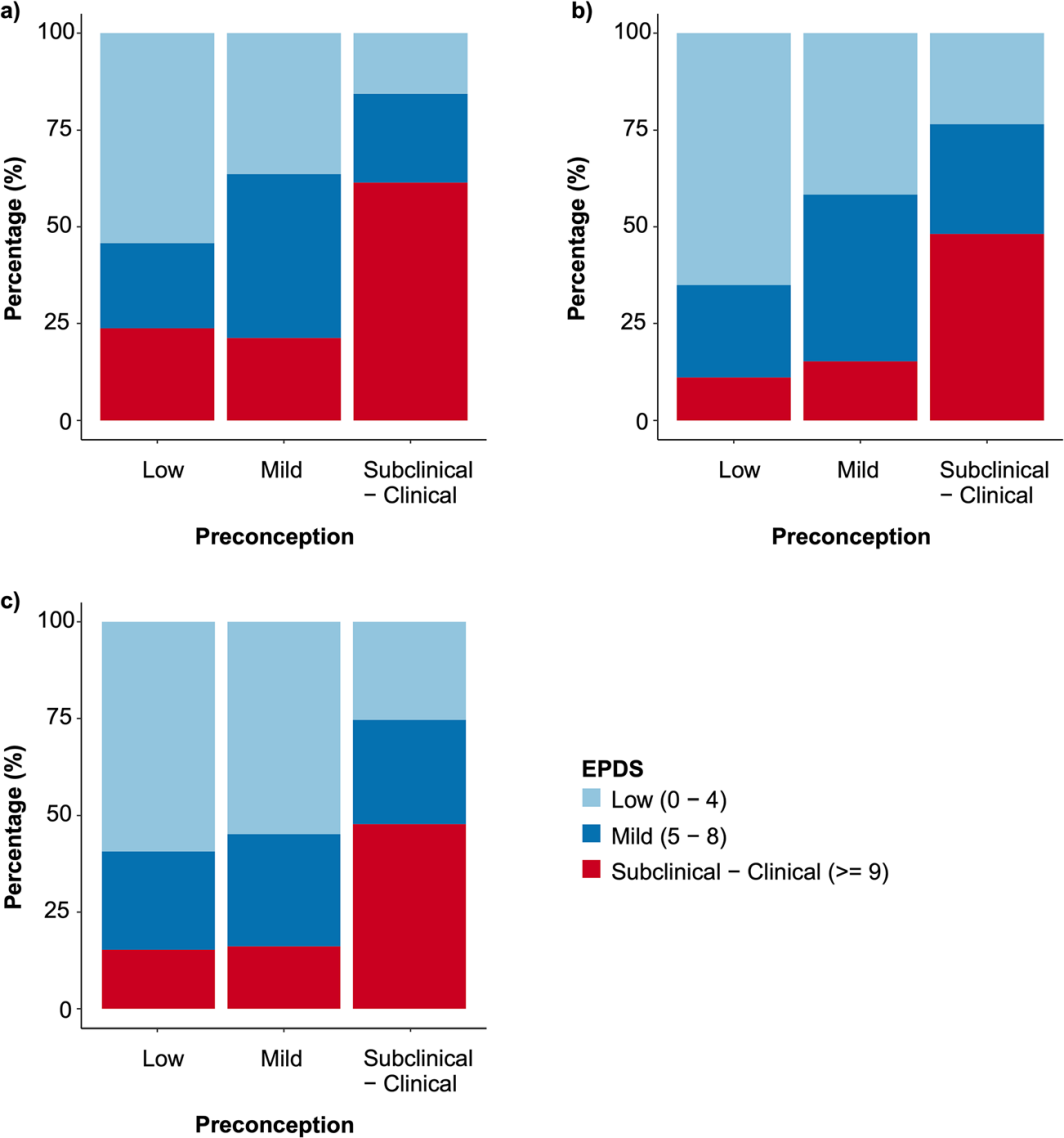
Bar-plots showing the progression of EPDS categories from preconception to **a** the first (PG1), **b** second (PG2), and **c** third trimester (PG3). Each column represents subjects in various EPDS groups during preconception, while y axis displays the percentage of women in the various EPDS groups during pregnancy

Visual inspection of the ICC’s presented in Fig. 2b shows that the values for the comparisons between the average preconception score and each of the three trimesters of pregnancy fell within the range of that for the individual preconception time points (i.e., the assessment of test-re-test reliability). A McNemar-Bowker’s test showed no significant difference in the EPDS categories between preconception and PG1 (*χ*^2^_(3)_ = 3.76, *p* = .29). The majority of women (52.8%) remained in the same EPDS category from preconception to PG1 (Fig. 3, Table 2). Importantly, the majority (61.4%) of women in the clinical/high-subclinical group at PG1 were classified as such during the preconception period. The variation that did occur from preconception to PG1 was bi-directional with 21% (*N* = 41) of women classified into more severe EPDS groups during PG1 than at preconception, while 26.1% (*N* = 51) improved in PG1 (i.e., were classified into milder EPDS categories).

**Table 2:** Number (%)of women in the various EPDS categories during preconception and subsequently in the first, second, and third trimesters

	First trimester (*N* = 195)				Second trimester (*N* = 216)				Third trimester (*N* = 188)			
Preconception	Low	Mild	Sub-clinical	Clinical	Low	Mild	Sub-clinical	Clinical	Low	Mild	Sub-clinical	Clinical
Low (0–4)	32 (54.2)	13 (22)	12 (20.3)	2 (3.4)	41 (65.1)	15 (23.8)	6 (9.5)	1 (1.6)	35 (59.3)	15 (25.4)	8 (13.6)	1 (1.7)
Mild (5–8)	24 (36.4)	28 (42.4)	12 (18.2)	2 (3)	30 (41.7)	31 (43.1)	7 (9.7)	4 (5.6)	34 (54.8)	18 (29)	9 (14.5)	1 (1.6)
Subclinical (9–13)	11 (19.3)	14 (24.6)	22 (38.6)	10 (17.5)	16 (24.2)	18 (27.3)	21 (31.8)	11 (16.7)	16 (30.2)	17 (32.1)	12 (22.6)	8 (15.1)
Clinical (≥ 14)	0 (0)	2 (15.4)	8 (61.5)	3 (23.1)	3 (20)	5 (33.3)	4 (26.7)	3 (20)	1 (7.1)	1 (7.1)	6 (42.9)	6 (42.9)

Percentages are calculated based on total number of cases in the different EPDS classes found during preconception period and are rounded off to the nearest 1 decimal place

Similar to PG1, 48.1% of clinical/high-subclinical in PG2 and 47.8% of those in PG3 were classified as such over the preconception period. The variation in assessment from preconception to PG2 and PG3 was also bi-directional with 15.3% and 18% of women worsening in category and 33.7% and 36.7% showing improvement. These changes are consistent with the general decline in symptoms across pregnancy (see above).

We next examined the correlation of the EPDS scores between preconception and 3-months postnatal. As expected, the scores between the time points had poor concordance (ICC = 0.39, 95% CI = 0.25–0.52). However, visual analyses of the ICCs between preconception and 3-months postnatal show that the ICCs remained within the range between average preconception score and during each trimester period (Fig. 2b). The difference in the EPDS categories between preconception and 3-months postnatal is not significant after post hoc analyses (*χ*^2^_(3)_ = 10.5, FDR *p > .05*). Importantly, similar to earlier observations, a high percentage (44.2%) of the women in the clinical/high-subclinical group at PN3 remained in the same group as during the preconception period.

### Network analyses

**Fig. 4:**
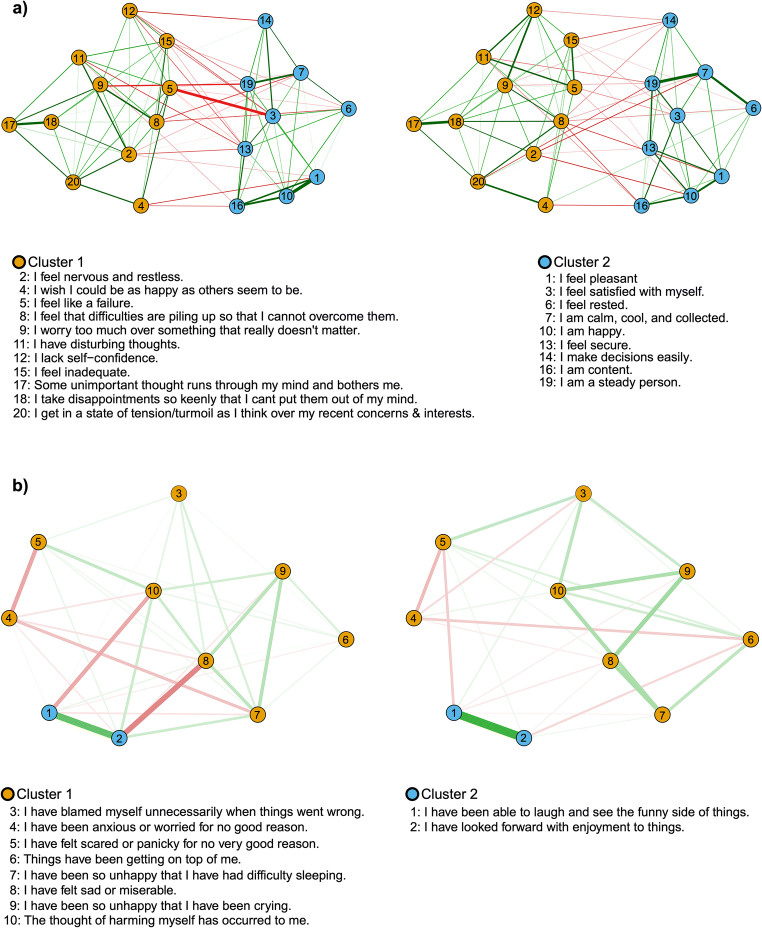
Fused graphical lasso networks of **a** STAI-trait and **b** EPDS, during preconception (left) and at the second trimester of pregnancy (right)

The analyses of scale scores revealed evidence for the stability from pre- to post-conception. We then asked whether this transition might be marked by a qualitative change in anxiety and depressive symptoms for which we used a symptom network analysis (Fig. 4a and b respectively). There were no significant differences between the structure of the preconception and prenatal anxiety (*p* = .71) and depressive networks (*p* = .43). There were also no significant changes in the global connectivity (i.e., absolute weights of the edges within the network) in the depressive (*p* = .21) and anxiety (*p* = .21) networks before and after conception.

**Fig. 5:**
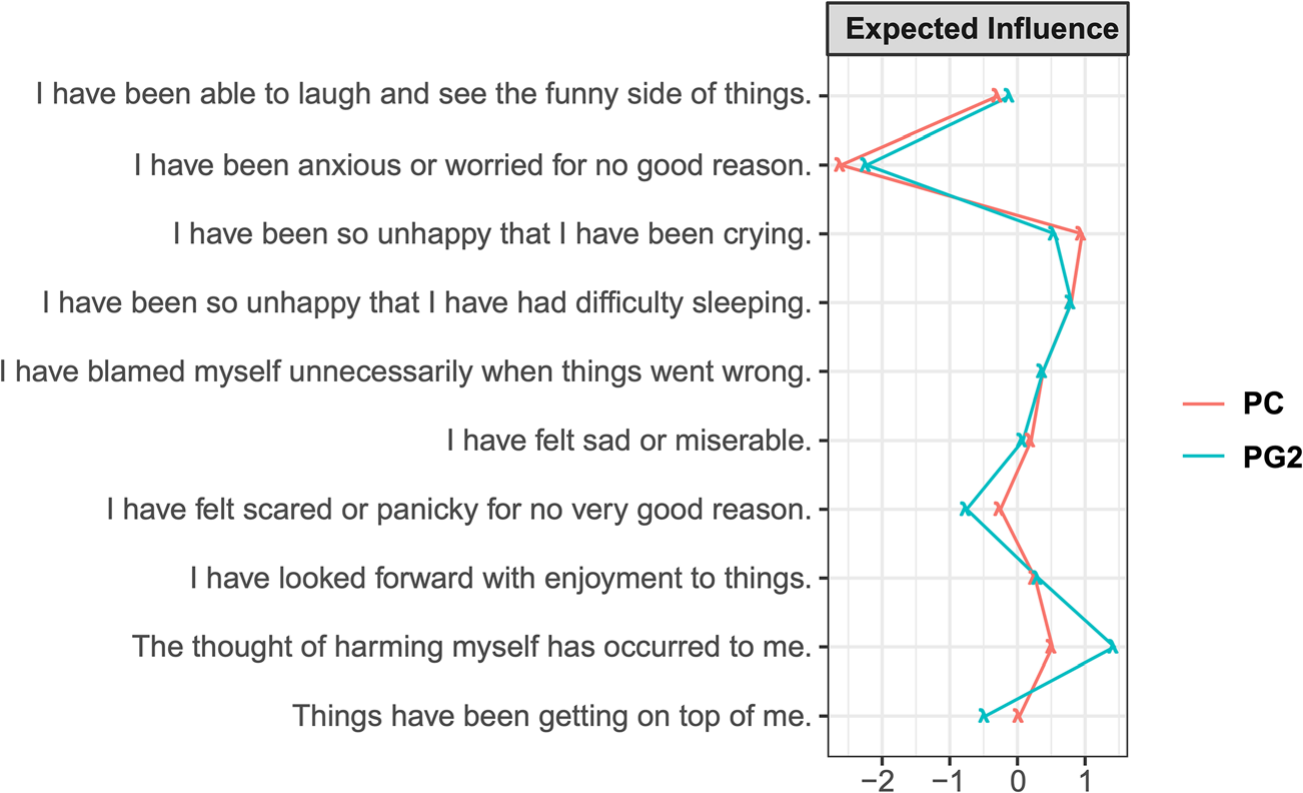
Expected influence (EI) indices of EPDS during preconception and the second trimester of pregnancy

**Fig. 6:**
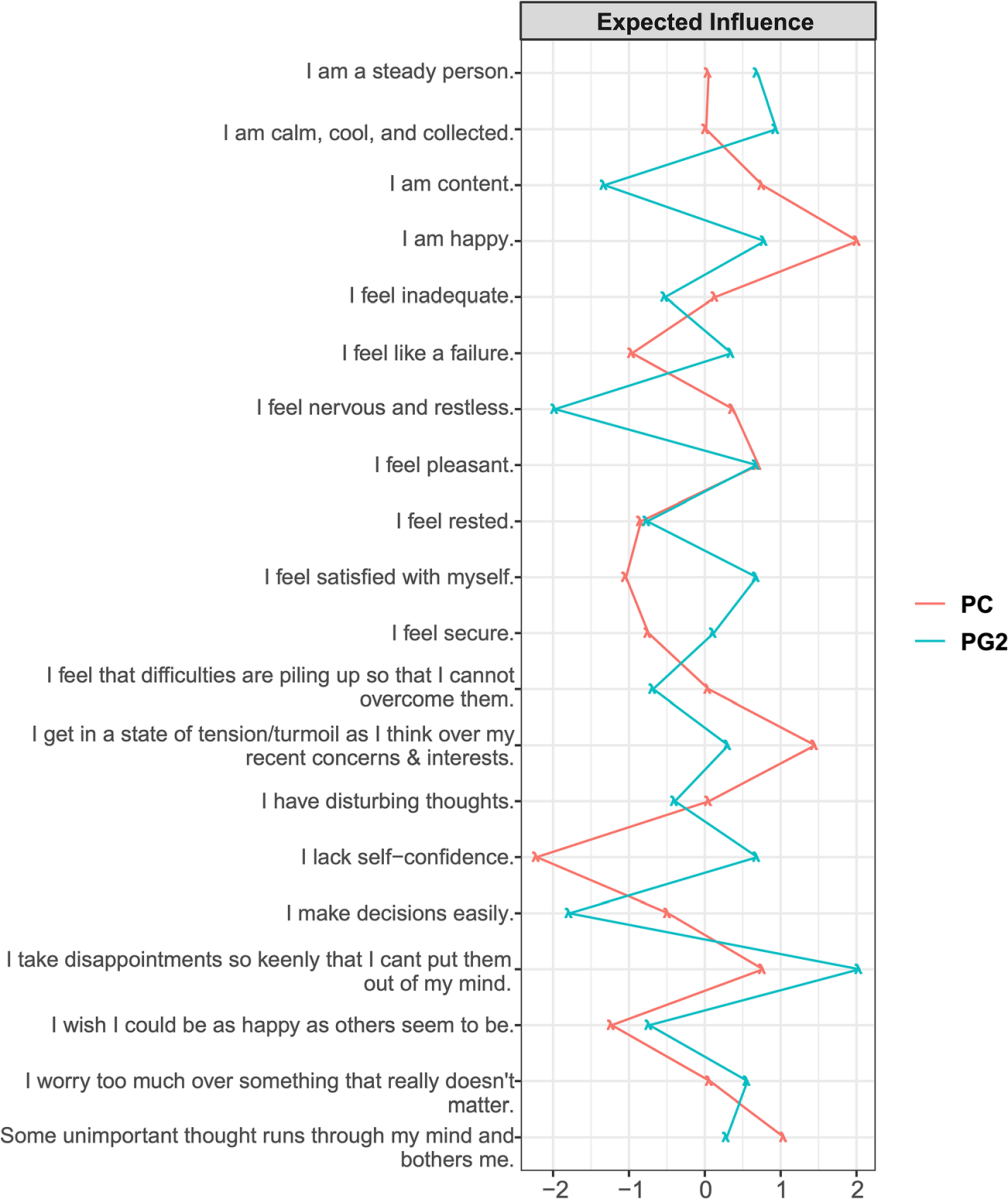
Expected influence (EI) indices of STAI-trait during preconception and the second trimester of pregnancy

The expected influence (EI) indices were stable and could be interpreted as the correlation stability coefficients were above the recommended threshold of 0.25 (Epskamp et al. 2018). The EI of the EPDS symptoms (Fig. 5) before and during pregnancy were temporally stable with a significant correlation of rs = .92, *p* < .001. According to the permutation-based test, none of the EI indices of the EPDS symptoms showed significant change (ps > .18). However, the EI of the STAI trait symptoms (Fig. 6) were not temporally stable with non-significant correlation of rs = .11, *p* = .64. The EI of “I-lack-self-confidence” was significantly different (difference = − 0.68, *p* = .005). The temporal difference in the EI of “I-lack-disappointment-keenly” approached significance (difference = −0.33, *p* = .056). The latter finding suggests some subtle variations are specific features of the anxiety network.

## Discussion

We used a preconception study to assess symptoms of depression and anxiety in women prior to and at multiple time points following conception using validated, self-report measures. The novel finding in our analysis is that of an impressive level of stability from the pre- to post-conception period for both symptoms of depression and anxiety. While this finding is consistent with previous studies showing that a prior history of depression strongly predicts the risk for perinatal depression (Leigh and Milgrom 2008; Giallo et al. 2014; Patton et al. 2015), much less is understood about the course of anxiety from preconception to antepartum period, possibly because antenatal anxiety remains poorly defined with no clear diagnostic categorization, although there exist studies of symptom patterns (Sinesi et al. 2019).

Importantly, our data suggest this level of continuity extends to symptoms experienced within a community sample. Likewise, our findings are consistent with earlier reports showing that, despite the substantial hormonal, psychological and social changes experienced from pregnancy to the postpartum period, individual differences in depressive symptoms remain stable over time. Thus, the strongest predictor of postnatal depression is depression in pregnancy (O’Hara and Swain 1996; Llewellyn et al. 1997). We also observed the previously reported pattern for a modest decline in symptom levels from the pre- to postnatal period (e.g., Evans et al. 2001; Heron et al. 2004). We note that there may be some remeasurement biases due to the decline in symptom levels. Our findings are comparable with previous epidemiological studies, notably Patton et al. (2015). In this study, 87% of pregnancies with reported onset of depressive symptoms during pregnancy had a preconception history of mental health problems. Similarly, 83% of pregnancies with post-partum depression had a preconception history of mental health problems. The similarities between our study and the Patton et al. study further support the notion that preconception mental health is a crucial predictor of antenatal and post-partum maternal mental health.

The critical finding presented in this report is that across our sample, the preconception levels of symptoms of depression or anxiety were effective predictors of both pre- and post-partum symptom levels. Our findings suggest that most women with high, subsyndromal, or clinical levels of depressive symptoms during pregnancy had experienced comparable levels of symptoms prior to conception. The same finding emerged for symptoms of anxiety. Our findings suggest that for many women, the mental health during pregnancy and following childbirth is generally continuous with the mental health status prior to conception. Previous studies indicate that poor preconception mental health predicts both poor antepartum (Witt et al. 2012) and postpartum mental health (Witt et al. 2011). This finding is also consistent with the idea that the most significant predictors of the risk for antenatal maternal depression (Milgrom et al. 2008; Biaggi et al. 2016), such as childhood adversity, low self-esteem, poor social support, and neuroticism, are generally stable influences operative over the periconceptual period.

Assessment of symptoms of depression or anxiety is commonly presented as scale scores that may not reflect more subtle, underlying features such as the relation between symptoms. We assessed this possibility by constructing and comparing symptom networks derived from the pre- and post-conception periods. The results reveal a striking level of similarity and imply little change in the relation between symptoms over the periconceptual period.

There are important implications for intervention. Women who report poor mental health before pregnancy are more likely to experience a pregnancy complication, to have a non-live birth, and to give birth to a low birth-weight baby (Witt et al. 2012). A remarkable longitudinal analysis (Spry et al. 2019) showed a significant increase in the percentage of offspring with high emotional reactivity born to mothers with persistent preconceptual mental health problems, and the bulk of this effect was statistically independent of later perinatal mental health status. These findings are consistent with other studies revealing that maternal stress imposed prior to conception associates with altered patterns of gene expression and behavior in the offspring (Miranda et al. 2011; Moog et al. 2016; see Keenan et al. 2018 for a review). Poor preconception mental health represents a vital, modifiable risk factor for poor obstetric outcomes with important influences on neurodevelopmental outcomes in the offspring. Accordingly, a life course perspective on maternal and child health suggests that interventions aimed at preventing adverse obstetric outcomes may be most effective if they begin in the preconception period (Lu and Halfon 2003). The results of previous prospective studies (Witt et al. 2011; Patton et al. 2015) discussed above suggest that the period for effective intervention extends earlier in life than the preconception period assessed in the current analyses.

Our study has both strengths and limitations. A strength is the dense data collection over the periconception period through to early postpartum. However, our sample sizes suggest that the data presented here should be considered preliminary and clearly merit replication in larger cohorts. Our use of a community sample is both a strength and limitation. The limitation is that our data cannot inform on more severe cases of depression or anxiety. However, the use of the community sample is important advantage since the influence of maternal symptoms of anxiety or depression on developmental outcomes for the offspring cuts across the population and is not unique to clinical cases (Meaney 2018). Moreover, the psychosocial impairments of women with high, subsyndromal level of depressive symptoms are comparable to those with clinical symptom levels (Judd et al. 1996; Weinberg et al. 2001). The other limitation of our study is that since we were primarily focused on data collection during pregnancy, we measured post-partum depression symptoms only at 3 months after birth. There could be more symptoms of depression within the first 6 weeks postpartum which were left undetected, since DSM-V classifies onset of peripartum depression within 4 weeks after delivery (American Psychiatric Association 2013). Additionally, the results of the network analyses in our study were limited to participants who conceived within a year after recruitment. As noted above, this is a limitation as some of the mental health problems may exist for many years beyond preconception (Patton et al. 2015) and is a case of concern for future predictive interventions.

## Conclusion

Our findings suggest that for most women, maternal symptoms of depression or anxiety remains stable from preconception into pregnancy. The symptom profiles of both depression and anxiety also remained virtually unchanged from preconception into second trimester. Our data thus imply that the appeal for preconceptual intervention should extend across the population and not only to clinical or high-risk cases.
